# Heritage language and linguistic theory

**DOI:** 10.3389/fpsyg.2015.01545

**Published:** 2015-10-09

**Authors:** Gregory Scontras, Zuzanna Fuchs, Maria Polinsky

**Affiliations:** ^1^Department of Psychology, Stanford UniversityStanford, CA, USA; ^2^Department of Linguistics, Harvard UniversityCambridge, MA, USA

**Keywords:** heritage linguistics, multilingualism, experimental methods, morphosyntax, syntax, semantics, pragmatics

## Abstract

This paper discusses a common reality in many cases of multilingualism: heritage speakers, or unbalanced bilinguals, simultaneous or sequential, who shifted early in childhood from one language (their heritage language) to their dominant language (the language of their speech community). To demonstrate the relevance of heritage linguistics to the study of linguistic competence more broadly defined, we present a series of case studies on heritage linguistics, documenting some of the deficits and abilities typical of heritage speakers, together with the broader theoretical questions they inform. We consider the reorganization of morphosyntactic feature systems, the reanalysis of atypical argument structure, the attrition of the syntax of relativization, and the simplification of scope interpretations; these phenomena implicate diverging trajectories and outcomes in the development of heritage speakers. The case studies also have practical and methodological implications for the study of multilingualism. We conclude by discussing more general concepts central to linguistic inquiry, in particular, complexity and native speaker competence.

## Introduction

Since its inception, the generative tradition within linguistic theory has concerned itself primarily with monolingual speakers in its quest for what we know when we know (a) language. The object of study, linguistic competence, or grammar, instantiates in and emerges from the brains of human speakers. Grammar cannot get loaded onto a microscope slide or set upon a scale; it gets accessed through its effects on naturally-developing speakers who employ the grammar in their native language *du jour*. Grammar informs and determines linguistic behavior; linguists study grammar by studying the behavior of speakers and making generalizations about the idealized state of mind of these speakers. But which speakers?

The investigation of grammar is necessarily a circuitous enterprise: we observe linguistic competence through linguistic performance, the situation-specific deployment of grammar. But extra-linguistic factors influence performance, so linguists help themselves to various domain restrictions in an attempt to limit noise in the translation from competence to performance. Chomsky (1965, p. 4) provides an early description of the obstacle to be overcome: “The problem for the linguist, as well as for the child learning the language, is to determine from the data of performance the underlying system of rules that has been mastered by the speaker-hearer and that he puts to use in actual performance.” Chomsky also provides an early characterization of one strategy for meeting this obstacle, focusing the linguist's attention on idealized, untainted language users:

Linguistic theory is concerned primarily with an ideal speaker-listener, in a completely homogeneous speech-community, who knows its language perfectly and is unaffected by such grammatically irrelevant conditions as memory limitations, distractions, shifts of attention and interest, and errors (random or characteristic) in applying his knowledge of the language in actual performance. (Chomsky, 1965, p. 3)

The rapid ascension of formal linguistics over the intervening five decades has demonstrated the success of this focused approach to the study of language (for a similar line of discussion, see Lohndal, 2013). A great deal of progress has been made to move beyond “grammars” in the traditional sense—comprehensive descriptions of language-specific regularities and their exceptions—to grammar in the Chomskyan sense: the rules and processes that generate those regularities in the first place.

Still, Chomsky's counsel necessarily excludes from study a wide swath of the world's language users, communities, and even languages. Put simply, the majority of speakers and speaking contexts fail to meet the admittedly idealized criteria above. But even ignoring the “grammatically irrelevant conditions” that govern the use of language, what do we make of the multitudes of speakers who may claim imperfect competence in more than one language? So far in the history of generative linguistics, the answer to this question has been “not much.” Citing the wealth of data that gets ignored in such an unrealistic exclusion, together with the unique questions these data stand to answer, Benmamoun et al. (2013b, p. 129) propose we augment our study of language by “shifting linguistic attention from the model of a monolingual speaker to the model of a multilingual speaker.” Similarly, Rothman and Treffers-Daller (2014) contend that multilingual speakers should be considered native in more than one language and call for a revision of the overall concept of a well-rounded native speaker. We follow these authors in focusing our attention on a subset of multilingual language users: heritage speakers.

To demonstrate the relevance of heritage linguistics to the study of language competence more broadly defined, this paper presents a series of in-depth case studies on heritage linguistics, documenting some of the deficits and abilities typical of heritage speakers. We adopt a modular approach to summarizing old and new findings, beginning with a look at the morphosyntax of agreement phenomena, then shift attention to the syntax of argument structure and of relativization; we then turn to the semantics and pragmatics of scope phenomena. The case studies we present serve double duty: first, their findings stand to characterize the similarities and differences between native and heritage speakers; and second, they engage with a popular strain of research in heritage language study, namely the various proposals meant to account for the near-native abilities of heritage speakers. Our aim is to show how the documented diversity of speaker profiles, abilities, and deficits requires a carefully nuanced approach to the study of multilingualism.

Before turning to the case studies, the remainder of this introduction describes the population of interest as it is typically characterized, together with various proposals meant to account for the unique linguistic competence of heritage speakers.

### Introducing heritage speakers

To illustrate the defining characteristics of a heritage speaker, we begin with a few hypothetical examples. For starters, meet Samantha. Her family is from Korea, but she was born in Los Angeles and has never traveled to Korea. While in Los Angeles, Samantha grew up immersed in the rich Korean culture that is prevalent there (Los Angeles has the largest Korean-American population in the USA). Samantha went to a Korean Sunday school when she was a child, and she still uses Korean with her family and at church. However, she is more comfortable speaking in English; and although she reads Korean, she prefers reading in English. Samantha is always rather nervous about her Korean not being good enough for her family.

Margot is only a hundred or so miles south of Samantha, living in a secluded area in La Jolla, California (outside of San Diego). Her family moved there from Russia when she was three, and her younger siblings were all born in La Jolla. Her father still has some business in Russia, but Margot and her siblings rarely go there. They prefer traveling to Western Europe, where everybody speaks English and they have an easier time communicating. When Margot and her siblings meet other Russians, they are always a bit suspicious of them and do not socialize too much.

Doris grew up in a Jewish family in the Bronx. All her friends were Dominican and Puerto Rican immigrants; she still keeps in touch with some of them, and readily switches back and forth between English and Spanish when they chat. Doris took Spanish in high school and quickly discovered that the language she learned from her friends was vastly different from the language in her textbook; she recalls the experience in her Spanish class as a nightmare. “Every time I spoke, my teacher mocked and belittled me for saying everything wrong. Apparently what was right for my friends was not right for the Anglo woman who was teaching me…”

Robert was born in Frankfurt, but when he was just a few months old, his family moved to Abu Dhabi, where his father worked as a banker. He had an Arabic-speaking nanny and went to an international school, but socialized with Arabic-speaking children (they all shared a passion in soccer). Robert moved back to Germany when he was 15, got his education in Germany, and is currently living in Berlin where he works as a graphic designer. He is still in touch with his friends in Abu Dhabi—they connect over social media—and it is his hope to save enough money to travel back to the place where he spent his childhood.

Shawn was born in Canada. His mother is Japanese and his father is British, fluent in Japanese. The family moved to Japan when Shawn was a toddler. He has received all of his education in Japanese, and although he has had a fair amount of English instruction and speaks English with his father now, as a young adult, he is more comfortable in Japanese. Recently, he took a course in American literature in his college; whenever possible, he tried to read the assigned books in a Japanese translation, which he found much easier than the original English.

What do these people have in common? They were all exposed to a certain language in their childhood, but then switched to another language, the dominant language of their society, later in their childhood. These are unbalanced bilinguals, sequential (Doris and Margot) or simultaneous (Robert, Shawn, Samantha), whose home language is much less present in their linguistic repertoire than the dominant language of their society. They may have gotten there in different ways, but they are all heritage speakers.

Narrowly defined, heritage speakers are individuals who were raised in homes where a language other than the dominant community language was spoken, resulting in some degree of bilingualism in the heritage language and the dominant language (Valdés, 2000). A heritage speaker may also be the child of an immigrant family who abruptly shifted from her first language to the dominant language of her new community. Crucially, the heritage speaker began learning the heritage language before, or concurrently with, the language which would become the stronger language. That bilingualism may be imbalanced, even heavily imbalanced, in favor of the dominant language, but some abilities in the heritage language persist.

Heritage speakers present a unique testbed for issues of acquisition, maintenance, and transfer within linguistic theory. In contrast to the traditional acquisition trajectory of idealized monolinguals, heritage speakers do not seem to exhibit native-like mastery of their first language in adulthood. As the definition of the heritage speaker makes clear, this apparent near-native acquisition owes to a shift of the learner's attention during childhood to a different dominant/majority language. However, the specifics of this attainment trajectory are anything but clear.

### Developmental trajectories of heritage speakers

The pathways to heritage speakerhood vary quite widely. Similarly diverse is the range of abilities that result. It should come as no surprise, then, that the proposed trajectories to the competence of heritage speakers are at least as complex as the speakers and abilities they are meant to characterize. Here we consider possible outcomes in the shape of heritage grammars. Setting aside the possibility that the heritage grammar can match that of the native baseline (something that we do not discuss in this paper, if only for lack of space), at least three other outcomes are possible: *transfer* from another grammar, *divergent attainment*, and *attrition* over the lifespan. Crucially, behavior with different grammatical phenomena may derive from diverging outcomes, owing in part to the broader linguistic context. Ultimately, research in heritage languages should be able to predict a particular outcome for a given phenomenon or context, but the field is not there yet. For now it suffices to survey the possibilities.

#### Types of outcomes

##### Dominant language transfer

An important point of contact between heritage speakers and second language learners lacking from traditional L1 acquisition is the interplay between the learner's first (heritage) language and second (dominant) language. Language transfer, or the nature of that particular interplay, is a foundational issue in second language acquisition research: to what extent does the first language grammar play a role in shaping the developing second language grammar? The effects of the native language on the acquisition of a second language in different levels of linguistic analysis (e.g., phonology, morphology, syntax, semantics, or the lexicon) have been extensively documented in the second language acquisition literature (e.g., Odlin, 1989; White, 1989; Gass and Selinker, 1992; Schwartz and Sprouse, 1996; Jarvis, 1998). The question of transfer arises in other language contact situations, including pidgin and creole genesis, where phenomena like lexical borrowings and so-called “areal features” are the well-known consequences of language contact. Research on bilingualism and language contact also suggests that the direction can reverse, such that the second language encroaches on the structure of the native language in systematic ways (Seliger, 1996; Pavlenko and Jarvis, 2002; Cook, 2003).

With the knowledge that grammar is a porous vessel whose contents are susceptible to contamination, in examining the linguistic characteristics of heritage grammars, the first question that often comes to mind is whether many of the “simplified,” non-standard characteristics observed in the heritage grammar could be due to transfer from the dominant language. For example, one can readily entertain the possibility that nominal and verbal inflectional morphology in Spanish and Russian heritage speakers gets eroded because the contact language in most of the heritage speakers tested to date is English, a language which does not mark gender on nouns or have rich tense/aspect and mood morphology. The same explanation goes for the preference for SVO word order over topicalization, which in turn leads to greater word order rigidity.

An obvious way to resolve this question over the source of simplified characteristics in heritage grammars is by testing heritage speakers whose majority language is typologically close to their heritage language (Spanish heritage speakers in Italy or Brazil, for example); ensuring that the contact language is at least as complex as the target language with respect to the phenomenon of interest controls for possible simplification transfer. Another option is to isolate the effects of different contact languages, either by comparing the effects of different dominant languages on one and the same heritage language, or by comparing the effect of one and the same dominant language on different heritage languages. In either case, one must take care to determine the status of the phenomenon of interest in *both* the heritage *and* the dominant grammar, to see whether there is anything to transfer in the first place. Put differently, comparison with a native speaker baseline does not suffice to prove transfer, as the native baseline might differ in important ways from its manifestation in the heritage population. We return to this cautionary tale below, and in our fourth case study, on scope calculations.

##### Divergent attainment

Heritage speakers are early bilinguals who learned their second (majority) language in childhood, either simultaneously with the heritage language, or after a short period of predominant exposure to and use of the minority language. A common pattern in simultaneous bilinguals is that as the child begins to socialize in the majority language, the amount of input from and use in the minority language is reduced. Consequently, the child's competence in the heritage language begins to lag, such that the heritage language becomes, structurally and functionally, the weaker language. Developmental delays that start in childhood never eventually catch up, and as the heritage child becomes an adult, the eventual adult grammar does not reach native-like development. This trajectory was originally introduced in the literature as “incomplete acquisition” (Polinsky, 2006; Polinsky and Kagan, 2007; Montrul, 2008; Benmamoun et al., 2013b); however, some researchers have argued against the use of this term because it has negative connotations (e.g., Pascual y Cabo and Rothman, 2012) or covers arguably unrelated phenomena, namely lack of mastery due to limited input vs. lack of knowledge associated with education and exposure to a standard dialect (e.g., Pires and Rothman, 2009). In this paper, we will be referring to the phenomenon as “divergent attainment,” in hopes that this term is more agreeable. Moving beyond the terminology, it is crucial to focus on contexts where such an outcome can be predicted; this is one of the larger goals of heritage language research.

A clear example of divergent attainment is the acquisition of the subjunctive in Spanish. Blake (1983) tested monolingual children in Mexico between the ages of 4 and 12 on their use of the subjunctive. He found that between the ages of 5 and 8, knowledge and use of the subjunctive was in fluctuation; children did not show categorical knowledge of the Spanish subjunctive until after age 10. Heritage speakers who received less input at an earlier age and no schooling in the language never fully acquire all of the uses and semantic nuances of the subjunctive, as reported in many studies (Silva-Corvalán, 1994; Martínez Mira, 2009; Montrul, 2009; Potowski et al., 2009; see also Silva-Corvalán, 2003, 2014, for longitudinal observations). It would seem, then, that the subjunctive employed by adult heritage speakers of Spanish evidences a calcified version of its attainment in monolingual youth.

##### Attrition

Distinct from, but not mutually exclusive with attainment is the outcome of attrition. Under normal circumstances, L1 attrition refers to the loss of linguistic skills in a bilingual environment. It implies that a given grammatical structure reached full mastery before suffering weakening or being subsequently lost after several years of reduced input or disuse. Thus, attrition is “the temporary or permanent loss of language ability as reflected in a speaker's performance or in his or her inability to make grammaticality judgments that would be consistent with native speaker monolinguals of the same age and stage of language development” (Seliger, 1996, p. 616). Attrition over the lifespan is a particularly intriguing case, since it challenges the common assumptions concerning the stability of structural change in adults.

Attrition often occurs during the first generation of immigration, affecting structural aspects of the L1 due either to language shift or to a change in the relative use of the L1 (De Bot, 1990)^1^. Attrition can also occur much earlier, having more dramatic effects on the integrity of the grammar. Recent research suggests that the extent of attrition is inversely related to the age of onset of bilingualism (Pallier, 2007; Montrul, 2008; Bylund, 2009; Flores, 2010, 2012). Prepubescent children tend to lose their L1 skills more quickly and to a greater extent than people who moved as adults and whose L1 was fully developed upon migration (Ammerlaan, 1996; Hulsen, 2000). That is, the extent of attrition and severe language loss is more pronounced in children younger than 10 or 12 years old than in individuals who immigrated after puberty. Research has also shown that severed or interrupted input in childhood, as in international adoptees, leads to severe attrition, including total language loss (Montrul, 2011).

There are two ways to tease apart divergent attainment and attrition in later childhood. The first strategy consists of conducting longitudinal or semi-longitudinal studies of children, like the ones by Anderson (1999), Merino (1983), and Silva-Corvalán (2003, 2014). These authors were able to document the incremental accumulation of errors in agreement (i.e., case or gender marking) in their investigation of immigrant children who arrived in their new country around age 8;0 or older. Their results show a significant accumulation of errors, which eventually leads to the loss of a baseline pattern. Still, it has yet to be determined at what point such error accumulation reaches the point of no return, resulting in severe language loss.

The other strategy for teasing apart attrition and divergent attainment compares children and adult heritage speakers. If it can be shown that normally-developing child heritage speakers perform better than their adult counterparts, then we have evidence for attrition. This strategy serves as the basis of our second and third case studies, which compare heritage speakers with monolingual controls, as well as with monolingual and heritage children.

#### What motivates the outcomes?

Having suggested three possible ways in which heritage language may differ from the baseline, we turn next to the potential sources for such differential outcomes. We explore three different scenarios: changes in the input, general constraints on memory, and universal structural principles.

##### Incipient changes in the input

To understand the source of seemingly non-native abilities in heritage language speakers, we must establish whether the immigrant communities themselves speak an altogether different variety from that spoken in the country where the language is dominant. In other words, it is important to ascertain patterns of language maintenance or change in the variety used by the immigrant community, to determine the input heritage language learners are receiving. Thus, one ought to determine whether the first generation grammar shows any of the non-standard properties attested in the heritage language; this approach is typical of sociolinguistic studies (Otheguy and Zentella, 2012). If the first generation grammar already shows signs of drift from the standard baseline, then the culprit is not the heritage learner. Conversely, if a property is not part of the register spoken to the heritage speakers, then it cannot be acquired, but must be the result of reanalysis or innovation.

To see the value in considering the grammar of first-generation immigrants in the shaping of heritage grammar, consider the findings of Montrul and Sánchez-Walker (2013), who tested differential object marking (DOM) in English-dominant heritage speakers of Spanish, first-generation immigrants (the input to the heritage speakers), as well as L1 speakers of different age cohorts in Mexico. The authors found that the child and adult heritage speakers omitted DOM, but so did the first-generation immigrants. The question then becomes: why did the input change in the first place? Answering this question brings us to two additional sources for the divergence between native and heritage grammars: general resource constraints (e.g., memory constraints) becoming more pronounced in a less dominant language, and universal structural properties of grammar extending their influence.

##### Resource constraints

Some changes in heritage language consist of constraining the domain within which a particular property applies. A recent example of this type of finding comes from Kim's (2007) study of binding interpretations by Korean heritage speakers in the USA and China. The study tested knowledge of binding interpretations with local and long-distance anaphors. Here we see deployed one of the suggestions made earlier for isolating the quality of transfer from a dominant language: comparing the effects of different dominant languages on one and the same heritage language. In many respects, Chinese and Korean are more similar than Korean and English. As such, Korean heritage speakers in China, who suffered less interference from their dominant language, were expected to be more accurate with long-distance binding than the Korean heritage speakers in the USA. However, Kim found that the two groups of Korean heritage speakers still had a marked preference for local binding, regardless of the contact language. Thus, the result state—loss of long-distance binding in heritage Korean—appears to have derived not from contact with a specific different system, but from contact with *any* different system. In other words, once the heritage language loses ground to another dominant language, whichever that language might be, resource-intensive phenomena like binding (or scope inversion; see Section *At the Interface: Scope Interpretations*) become more restricted.

The loss of long-distance binding in heritage Korean appears to be an instance of general constraints on memory becoming more pronounced in heritage speakers: shorter dependencies are preferred because they make fewer demands on the parser's memory. Given that the heritage speaker is already performing the costly task of speaking in a less dominant language, the cost of resource-intensive operations explodes, sometimes to the point of totally obscuring the availability of the operation.

##### Universal principles of language structure

In heritage grammars, where speakers are limited in their deployment of complex grammatical phenomena, language structure sometimes follows what looks like a default design, employing a seemingly restricted set of grammatical categories and operations. The list of default-like structures attested for heritage languages includes the use of dependencies which target only the highest structural constituent (as in the Russian relativization discussed in Section *Relativization: In Support of Universal Structural Principles*); the absence of nesting dependencies (Benmamoun et al., 2013a,b); the elimination of irregular morphology and the concomitant rise of analyticity (Benmamoun et al., 2013a,b); rigid word order (Isurin and Ivanova-Sullivan, 2008; Ivanova-Sullivan, 2014), often accompanied by the placement of closely associated items next to each other, in keeping with Behaghel's First Law (Behaghel, 1909; Haiman, 1983); and the lack of non-compositional structures (Dubinina, 2012; Rakhilina and Marushkina, 2014). All of these properties appear to at least superficially make the heritage language more user-friendly, in accord with general properties of language structure.

However incomplete, this list of properties bears a striking similarity to recurring traits observed in creole languages and often associated with the underlying innate principles of language structure, as in Bickerton's famous Bioprogram (Bickerton, 1984, 1988). We are not trying to propose a new version of the Bioprogram here, but we would like to offer two considerations. The first one is obvious: since there appear to be recurrent features observed in heritage language, a comprehensive list of heritage-language-specific properties related to universal principles of *optimal* language design is needed. Such a list needs to be established empirically, on the basis of a larger set of studies, and then re-evaluated in light of linguistic theory. Doing so would allow us to understand in a more coherent way the notion of language defaults and optima. Relatedly, given the initial evidence for their reliance on universal language principles, heritage speakers have a great deal to offer linguistic theory, because they speak directly to Plato's problem in language: showing how a grammar can be acquired under conditions of reduced input and usage. This reality makes heritage languages a desirable object of investigation, and we need to learn how to use them better to enrich the debate about the nature of the language faculty.

This completes our brief introduction to the population we herewith study: heritage language speakers. A reader interested in more details of this group can find further discussion in Benmamoun et al. (2013a,b), Montrul (2008) and Polinsky and Kagan (2007). In the remainder of this paper, we examine in considerable detail specific properties of heritage language grammar through a series of case studies. In doing so, we pursue two interconnected goals. First, we present theoretically relevant phenomena whose status in heritage language serves as evidence for a particular trajectory or outcome, either contrasting with the native baseline (as with morphosyntax in Section *Agreement Morphology and Category Structuring*) or in support of general structural principles (as with syntax in Sections *Argument Structure: The Unaccusative Challenge* and *Relativization: In Support of Universal Structural Principles*). Second, by concentrating on areas of known vulnerability in language structure, we show that the ultimate fate of vulnerable domains can vary depending on the level or type of representation and its specific language context.

We begin our investigation with a look at morphosyntax, agreement in particular (Section *Agreement Morphology and Category Structuring*). We then analyze phenomena related to argument structure (Section *Argument Structure: The Unaccusative Challenge*) and syntactic dependencies (Section *Relativization: In Support of Universal Structural Principles*). In Section *At the Interface: Scope Interpretations*, we venture outside narrow syntax and consider the grammar of scope, which brings together several interfacing grammatical domains. Section *Conclusions* presents our conclusions, where we revisit the question of what it means to be a native speaker, and what linguists stand to gain from embracing the reality of heritage linguistics.

## Agreement morphology and category structuring

In our first case study, we extend previous work on the morphosyntax of agreement in Spanish. Given the well-documented difficulty heritage speakers display with morphology in general and agreement morphology in particular (see Benmamoun et al., 2013b, pp. 141–144, and further references therein), we expected to find differences between native and heritage speakers of Spanish, and, more importantly, we expected these differences to be informative with respect to the agreement mechanism and its features in these minimally-differing grammars. But before asking how heritage speakers of Spanish perform, we must first establish the native baseline.

In Fuchs et al. (in press), we investigated the organization of number and gender features in Spanish, bringing experimental evidence to bear on the structure and content of agreement. The choice of number and gender features was not accidental: the third class of agreement features, person, stands apart both descriptively (for example, unlike the other features, person agreement never appears on adjectives; see Baker, 2008) and theoretically (cf. the hierarchical positioning of person in the feature geometry of Harley and Ritter, 2002). Meanwhile, the relationship between gender and number is less clear. Assuming that both features are represented in syntax, there are two analytical possibilities, both proposed in the literature. According to one scenario, gender and number are always bundled together (cf. Ritter, 1993; Carstens, 2000, 2003). Under the bundling model, number and gender features are projected and valued together; the valuation of gender presupposes a valuation of number, as gender features do not project independently of number. The bundling model draws its empirical inspiration from the fact that languages regularly combine gender and number information in the morphology; one rarely finds systems where the two features participate in agreement and yet are independent of each other.

In the alternative, split model (Picallo, 1991; Antón-Méndez et al., 2002; Carminati, 2005), gender morphology hosted on a nominal stem heads its own syntactic projection (GenP), and GenP is dominated by NumP (i.e., the source of number features/morphology). Thus, number and gender features are projected—and therefore also valued—independently of each other. One of the major arguments in favor of the split model comes from the order of morphemes in nominal derivations. In those languages where number and gender morphology can be descriptively separated, the order is Stem-Gender-Number, as in the following Spanish examples:

(1) a. [[*libr*]-[_GenP_ *o*-] [_NumP_ *s*]] ‘books’
b. [[*libr*]-[_GenP_ *o*-] [_NumP_ ø]] ‘book’

Because it levels the hierarchical distinction between number and gender, the bundling model does not have a straightforward way of predicting the ordering in (1). That the split model derives such an order is a side effect of the simple feature geometry: number dominates gender^2^. But which model, bundling or split, is the right one for Spanish? This was the question we set out to answer in Fuchs et al. (in press).

In Spanish, number and gender are expressed through independent suffixes. For gender, the word marker *-a* most often corresponds to the feminine, and the word marker *-o* most often corresponds to the masculine (although see Harris, 1991, for a more detailed discussion and many exceptions). Number is represented much like it is in English: The plural is marked by *-s*, whereas the singular receives no marking. Determiners and adjectives must agree with the noun in both number and gender.

(2) a.    la                manzana     b.   el               plátano
        the.f.sg       apple.f.sg         the.m.sg     banana.m.sg
 c.    las               manzanas   d.    los             plátanos
        the.f.pl       apple.f.pl          the.m.pl     banana.m.pl

As the number and gender agreement morphemes are in principle independent, we could manipulate their combination to produce sentences with different kinds of agreement errors in the Fuchs et al. study. Because the bundling and split models of feature geometry make different commitments regarding the valuation of agreement features, the predictions of the two models pull apart in cases of agreement attraction. In such cases, like the English example in (3), a noun (italicized) intervenes between the head noun (underlined) and its predicate (in bold), and the predicate incorrectly enters into agreement with the intervening noun rather than the head noun (in (3), *were* is plural, but should be singular to match the number of the head noun *key*). Because features of the local noun match features of the predicate, people incorrectly perceive the sentence as grammatical. This is agreement attraction.

(3) The key to the *cabinets* **were** lost.

Cases of agreement attraction have been experimentally studied in various languages, testing whether there is an asymmetry between different values of features in triggering agreement errors (e.g., English: Bock and Miller, 1991; Bock and Eberhard, 1993; Vigliocco et al., 1996; Vigliocco and Nicol, 1998; Bock et al., 2012; Spanish: Vigliocco et al., 1996; Antón-Méndez, 1999; Antón-Méndez et al., 2002; Alcocer and Phillips, 2009; Lago et al., 2015; Italian: Vigliocco et al., 1995; Vigliocco and Franck, 1999; French: Vigliocco et al., 1996; Dutch: Bock et al., 2001; Dutch and German: Hartsuiker et al., 2003; Russian: Lorimor et al., 2008). In Fuchs et al., we extended the method by putting the phenomenon of attraction to use in exploring the difference between bundling and split approaches.

Recall that if number and gender are bundled, then they ought to be valued simultaneously. This suggests that the number and gender features of a noun should determine agreement together, at the same time. When an incorrect noun enters into agreement with an adjective, both its number and gender features should effect agreement attraction. To illustrate this point, consider the following ungrammatical sentences:

(4) a.   *El                  niño                      considera                      la                      noticia
            the.m.sg         boy                      consider.pres.3sg         the.f.sg             news.item.f.sg
            en los             periódicos             terriblemente                aburridos.
            in the.m.pl     magazine.m.pl       terribly                          boring.m.pl
            (‘The boy considers the news item in the magazines to be terribly boring.’)
      b.   *El                 niño                      considera                       la                      noticia
             the.m.sg        boy                      consider.pres.3sg          the.f.sg             news.item.f.sg
             en                  las                        revistas                          terriblemente     aburridas.
             in                   the.f.pl                magazine.f.pl                 terribly              boring.f.pl
             (‘The boy considers the news item in the magazines to be terribly boring.’)

Both (4a) and (4b) are ungrammatical. However, in each sentence the local noun has entered into agreement with the adjective, which may lead to an illusion of grammaticality via attraction. If number and gender are projected and valued together, per bundling approaches, then when the probe (incorrectly) gets a feature (e.g., number) from the local noun, it should be able to get the other feature (e.g., gender) as well. In other words, agreement attraction in one feature ought to precipitate agreement attraction in the other feature, with the result that both of the above sentences should be rated equally high (or equally low).

If, however, number and gender are split, then they are projected and valued independently, and agreement attraction in number can proceed independently of agreement attraction in gender. This means that, all other factors being equal, a violation in gender agreement may be judged higher or lower than a violation in number agreement. Crucially, the violations are evaluated on their own merits. Furthermore, if the two features are independent of each other, we can expect that a violation in both of them would be more offensive to a comprehender than a violation in just one feature. This expectation is based on the observation that the more grammatical constraints violated, the higher the degree of degradation (consider Kluender, 2004). Applying that logic, we expected that the violation in (4a), where both the gender and the number of the head noun are mismatched, should be rated lower than (4b), where only the number feature is mismatched. Thus, under a split model, (4a) should receive a lower rating.

**Figure 1:**
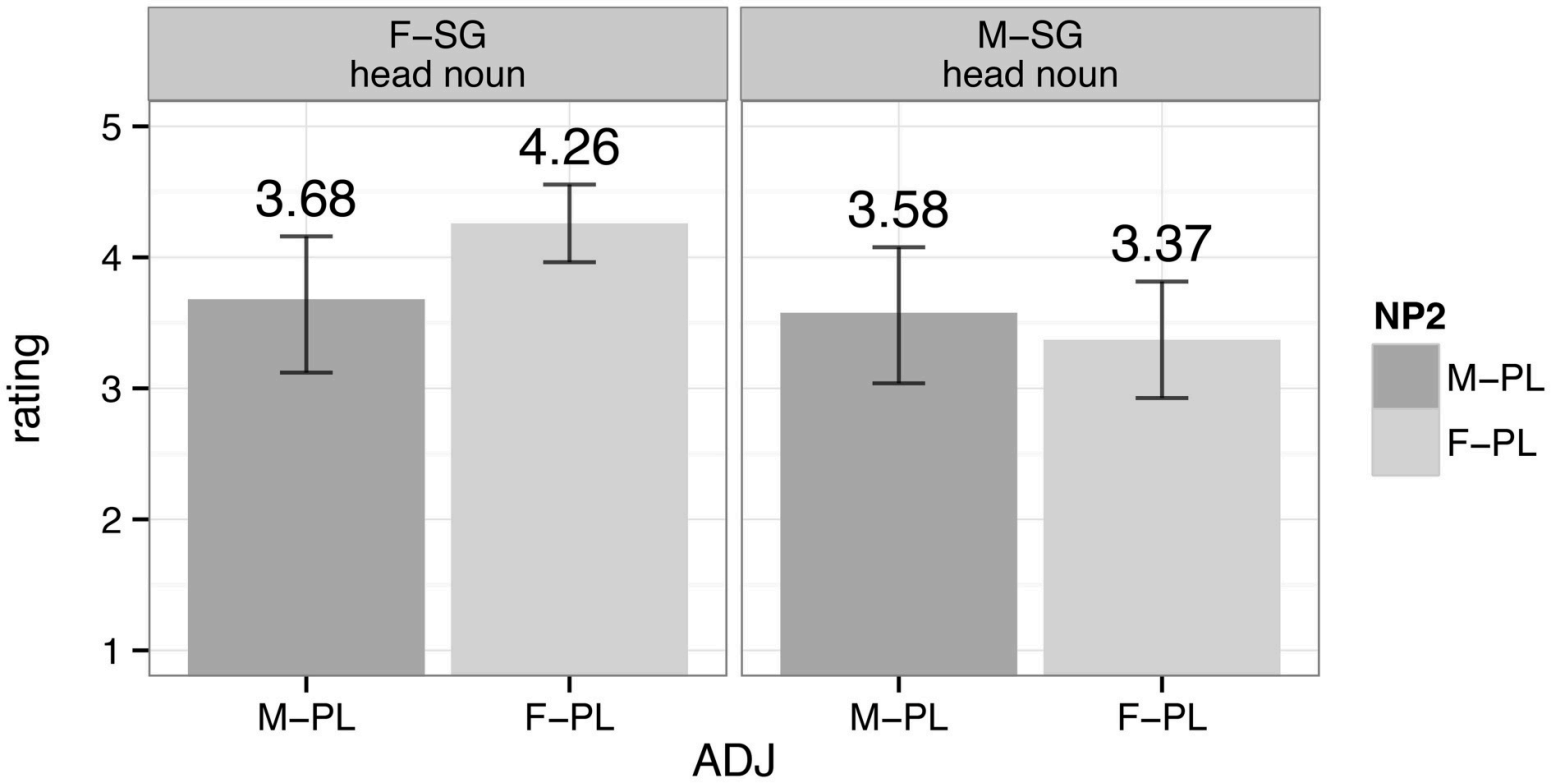
**Average ratings for potential attraction conditions (Fuchs et al., in press)**.

We originally tested native speakers of Spanish (*n* = 50) in an auditory sentence-acceptability rating task involving sentences as in (4), with differing numbers of agreement errors. In each of these critical conditions, the head noun appeared in the singular while the local noun and adjective appeared in the plural. By permuting the gender of the head noun, the local noun, and the adjective, we engineered potential attraction conditions in which the local noun either agreed with the adjective in only number (i.e., both were plural, but their gender did not match), or in both number and gender. Participants heard a recording of the sentence, and then were asked to rate its acceptability on a 5-point Likert scale (1 = “completely unacceptable”; 5 = “completely acceptable”). The results are plotted in Figure 1, which organizes ratings by potential attraction condition; error bars represent bootstrapped 95% confidence intervals drawn from 10,000 samples of the data.

For feminine head nouns, the sequence with a single agreement error, **F.SG**—F.PL—F.PL, was rated significantly higher than the sequence with two agreement errors, **F.SG**—M.PL—M.PL^3^. Thus, we found evidence of attraction such that ungrammatical sequences were accepted, but attraction occurred only between the number features of the local noun and adjective; if the gender of the head noun did not match that of the adjective, the sentence was correctly viewed as sub-par. For masculine head nouns, the difference between ratings given for single-error attraction conditions (**M.SG**—M.PL—M.PL) and double-error attraction conditions (**M.SG**—F.PL—F.PL) was not significant; we failed to find evidence of attraction at all for masculine head nouns.

Given the predictions of the bundling vs. split models, we interpreted the asymmetry in the ratings of agreement mismatches for feminine head nouns as evidence that number and gender features are valued separately; were they valued together, we should have found no difference between the conditions in which only one feature determined attraction effects and the conditions where both features caused attraction. Thus, in Spanish, a split model of number and gender features best accounts for the data: these features are treated separately in agreement.

**Figure 2:**
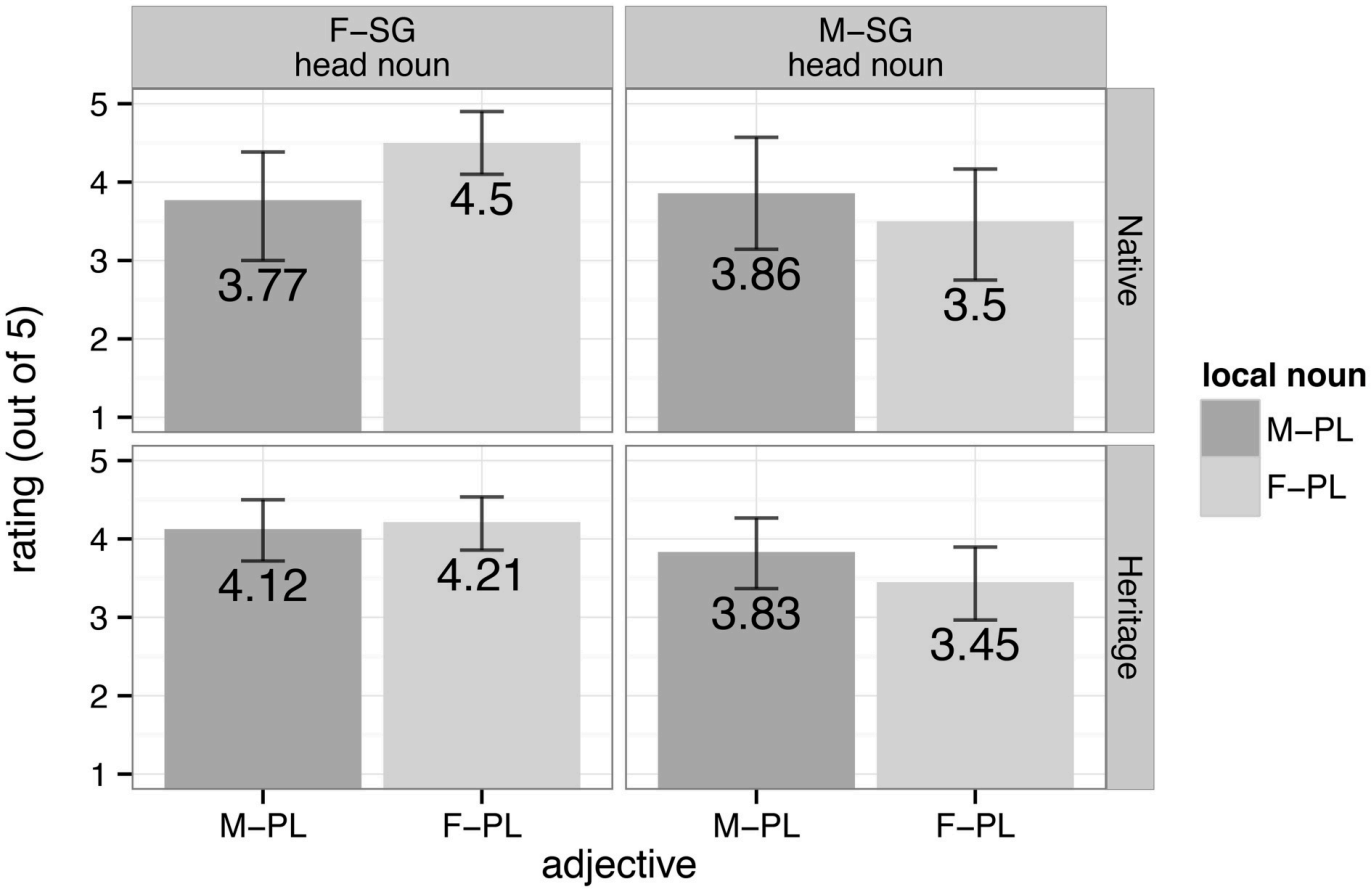
**Average ratings for potential attraction conditions from native (top) and heritage (bottom) speakers of Spanish in an extension of the methodology from Fuchs et al. (in press)**.

Now, given the precarious status of agreement morphology in heritage grammars, our question shifts to whether heritage speakers diverge from native ones in their agreement behavior, such that their representation of number and gender features is fundamentally different from the baseline. We extended the auditory sentence-acceptability rating task from Fuchs et al. to English-dominant heritage speakers of Spanish, as well as baseline controls. The results appear in Figure 2.

Note first that the results of our new population of native speaker controls (*n* = 28) replicate those found in the original study: participants perceived conditions with agreement errors in both number and gender as ungrammatical and rated them lower than conditions with an agreement error in only one feature; and feminine vs. masculine head nouns were treated differently.

Turning to heritage Spanish, we identified these speakers on the basis of a demographics questionnaire that preceded testing. Heritage speakers (*n* = 71) were those who indicated that they first learned Spanish and then English, had no formal education in Spanish, and who never lived in a Spanish-speaking country during childhood. Figure 2 shows that heritage speakers behave similarly to the native baseline in treating feminine vs. masculine head nouns separately with respect to attraction. However, unlike native speakers, heritage speakers rated attraction conditions equally high, regardless of the number of agreement mismatches between the head noun and the adjective. As long as the attractor noun agreed with the adjective in at least one feature, attraction succeeded and participants rated these ungrammatical sentences as acceptable.

The most straightforward interpretation of these results, in accordance with our original predictions for the native baseline, would have heritage speakers bundle number and gender features so that they are projected and valued together. However, before jumping to this conclusion, we must be realistic about the morphological limitations in heritage language, limitations that motivated the current study in the first place. What if the observed insensitivity to the number of agreement errors signaled not that number carries gender along for the ride while it gets valued in the heritage grammar, but rather that our heritage participants did not access gender as they processed the data presented to them? In other words, it could be the case that our heritage speakers simply ignored gender altogether. While we lack conclusive evidence to tease apart bundling from ignorance (i.e., from the ignoring of gender), the differential treatment of feminine vs. masculine head nouns in accord with the native baseline suggests that at least at some level, heritage speakers are attending to gender. If we take this evidence seriously, then heritage speakers have reanalyzed the feature system of Spanish so that it levels the hierarchical distinction between number and gender. Put simply, what native speakers treat as separate categories (i.e., number and gender), heritage speakers handle as but one, thus opting for the bundling of these categories. The result is a different, ostensibly simpler grammar than that of the baseline.

## Argument structure: The unaccusative challenge

Having considered differences in the domain of morphosyntax, we now leave the “morpho” component behind and dive head-first into syntax. But which syntactic phenomena might undergo change in heritage languages? Atypical, complex, or infrequent constructions prove particularly difficult to master in monolingual L1 acquisition. These structures, which stand on unsteady footing already in the native baseline, ought to be particularly vulnerable to reanalysis in heritage grammars. Thus, they are excellent candidates for the study of syntactic differences between monolingual and heritage speakers.

Bearing this vulnerability in mind, Pascual y Cabo (2013) targeted Spanish psych-verbs in a processing study that compared native and heritage, adult and child grammars. Cross-linguistically, psych-verbs denote a mental or emotional state, or the process that leads to such a state. These verbs are not uniform (e.g., Belletti and Rizzi, 1988; Landau, 2010); in Spanish, they fall into at least three classes. Pascual y Cabo concentrates on Spanish class III psych-verbs, among which *gustar* “like” is the most common. These psych-verbs are also referred to as reverse psychological predicates (RPP), owing to their non-standard argument mapping: the experiencer precedes the verb [*Katherine* in (5a)], but it is the post-verbal theme [*los kiwis* in (5a)] that is the syntactic subject of the sentence. Verbs of this type necessarily receive a stative reading. As strict statives, they expectedly resist passivization, as in (5b); syntactic accounts tie the lack of passivization to the absence of an agent-introducing *v*P projection in their argument structure (Belletti and Rizzi, 1988). Other classes of psych-verbs, namely those that allow agentive readings like *molestar* “bother” in (6a), can be passivized, (6b).

(5) *Spanish class III psych-verbs*
     a.     A            Katherine     le                 gustan                 los              kiwi-s.
             to            K.               3sg.dat.cl   like.prs.3pl       the.m.pl      kiwipl
            ‘Katherine likes kiwis.’
     b.    *Los        kiwis            son              gustad-os            (por           Katherine).
            the.m.pl   kiwipl          be.prs.3pl   like.ptcp-m.pl    by             K.
            Intended: ‘The kiwis are liked by Katherine.’

(6) *Spanish class II psych-verbs*
     a.     Diana     molestó                         a Adam.
             D.          bother.pst.3sg              to A.
            ‘Diana annoyed Adam (intentionally).’
      b.    Adam     es                                 molestad-o          (por          Diana).
             A.           be.prs.3sg bother.     ptcp-m.sg           by            D.
            ‘Adam is annoyed by Diana.’

This argument structure of stative psych-verbs has been the subject of much discussion in the literature on L1 and L2 acquisition of Spanish. Gómez Soler (2011) analyzes spontaneous child speech and shows that children start producing target-like *gustar* constructions quite early, at approximately age 1;10. In a subsequent comprehension study, Gómez Soler (2012) determined that children as young as 3-years-old are able to comprehend this class of psych-verbs, but children's performance varied according to the specific verb used. Children performed remarkably well (at 79% accuracy) with *gustar*, but at chance (52%) with less common stative-only psych-verbs like *faltar* “lack.” As is so often the case, different tasks yield different findings: a different comprehension study by Torrens et al. (2006) argued that children do not have adult-like understanding of these psych-verb constructions until around age 6;0. Although the exact time of acquisition of stative-only psych-verbs in Spanish is still up for debate, the evidence at hand supports the modest claim that they are acquired later by monolingual Spanish children than agentive predicates with regular argument-theta-role mappings.

Moving away from the native baseline, it should come as no surprise that these constructions also prove difficult for less idealized populations of learners. Regardless of the L1 of the speakers tested, psych-verbs with atypical argument structure consistently prove difficult for L2 learners of Spanish (Montrul, 1997; Quesada, 2008), although L2 learners eventually attain L1-level competency in producing and comprehending such constructions. With these facts in mind, Pascual y Cabo shifts attention to English-dominant heritage speakers of Spanish, who often lack formal schooling in their less dominant language. He notes that psych-verbs like *gustar* have two properties that make them vulnerable in the heritage grammar: their atypical argument structure, and the relative difficulty of their L1 acquisition. Based on a comprehension study of class III psych-verbs in Heritage Spanish, Pascual y Cabo hypothesizes that heritage speakers of Spanish reanalyze the psych-verb *gustar* to be optionally agentive, rather than strictly stative. In other words, heritage speakers might mistakenly align the argument structure of stative-only psych-verbs with less exotic agentive psych-verbs like *molestar*.

If this reanalysis were to take place, we should find evidence of it in passive constructions; this is precisely what Pascual y Cabo investigated. He predicted that if class III psych-verbs get reanalyzed as class II psych-verbs in heritage grammars, then heritage speakers would accept *gustar* and other such verbs in passive constructions. Native speakers, however, would find these constructions invariably unacceptable. The results of his acceptability judgment task confirmed this prediction: as expected, native speakers found passive constructions for stative-only psych-verbs to be categorically unacceptable, while heritage speakers at varying levels of proficiency rated these constructions as more acceptable. Pascual y Cabo argued that this result was sufficient to confirm his hypothesis that heritage speakers find *gustar* to be more compatible with passive constructions than native speakers do, and that this compatibility evidences the fact that heritage speakers are at least sometimes reanalyzing stative class III psych-verbs as agentive. Pascual y Cabo then considered the possible trajectory of this reanalysis. In order to determine whether the outcome implicated attrition, divergent attainment, or some other factor, Pascual y Cabo compared the performance of the original population of adult heritage speakers to child heritage speakers and child monolingual speakers, using the same acceptability task.

If the reanalysis of *gustar* were due to attrition, then at some earlier point in the lifespan of heritage speakers we would find more target-like behavior, which was lost on the way to adulthood (recall the discussion in Section *Developmental Trajectories of Heritage Speakers* above). Concretely, we would expect monolingual (and heritage) children to perform better at correctly judging passive *gustar* constructions to be unacceptable. However, this was not the case: both monolingual and heritage children performed worse than the adult heritage speakers. The fact that adult heritage speakers behave more like adult native speakers than do child monolingual speakers suggests that heritage speakers do improve their performance with these psych-verbs over time, and thus that the observed reanalysis does not arise from attrition. This improvement likewise suggests that divergent attainment is not the cause of reanalysis. Under a divergent attainment story, we would expect similar behavior between child and adult heritage speakers.

Following Lightfoot (1991, 1999, 2012), Pascual y Cabo argues that “superficial performance innovations provided in the input from the immigrant generation contribute to the changes in H[eritage] S[peakers'] grammars” (Pascual y Cabo, 2013, p. 131). The original source, then, is attrition among L1 monolingual immigrants, who sometimes produce target-like *gustar* constructions, and sometimes do not. Next generation immigrant speakers (i.e., heritage language learners) receive this already non-standard input from their parents, which results in ambiguity in their mental representations of the syntax of the constructions at issue. The ambiguity forces heritage speakers to (economically) reanalyze the constructions, delivering the otherwise off-limits agentive constructions for psych-verbs.

The treatment of psych-verbs in heritage Spanish is clearly an innovation, the seeds of which are present in the native baseline, where verbs with non-canonical argument structure show a certain degree of instability. While it is clear that L1 speakers of Spanish ultimately acquire affective (experiencer) verbs, or at least *gustar*, the most prominent and frequent one among them, there are some Spanish dialects, for example in South America, where experiencers are expressed as subjects (not indirect objects; Anagnostopoulou, 1999); and there are other dialects where experiencers are encoded as direct objects (Franco, 1993, 1994). This variation indicates a certain degree of instability in the experiencer marking, exactly the instability that Pascual y Cabo picks up on in his description of the heritage speaker input. In addition, all heritage speakers of Spanish surveyed by Pascual y Cabo were dominant in English, which lacks similarly quirky subjects. Thus, even structural transfer from English may not be off the table as a possible contribution to reanalysis in these heritage speakers. Could we ever find instances of genuine reanalysis in adult heritage speakers, without transfer effects? We contend that such reanalysis is possible, and we turn to its example in the next section.

## Relativization: In support of universal structural principles

Long-distance dependencies, relative clauses in particular, have long attracted the attention of linguists because they offer a window onto structural preferences in languages. If a language can relativize at a given position in the accessibility hierarchy in (7), then it can relativize at every position above it. To illustrate, if a language allows relativization of the oblique object, then we can expect the language to also allow relativization of the indirect object, direct object, and subject; if a language only allows one kind of relative clause, it will be a subject-extracted relative clause. Relative clauses also offer an excellent test case of memory constraints, which the parser needs to reckon with in the formation of long distance dependencies between the filler and its gap.

(7) *Accessibility hierarchy* (Keenan and Comrie, 1977)
subject > direct object > indirect object > oblique object >
possessor > standard of comparison

Consider the subject-extracted relative clause in (8a), and the object-extracted relative clause in (8b). In both cases, the gap and the relative pronoun reference the subject of the matrix clause, *the reporter*.

(8) a. The reporter_*i*_ who_*i*_ _____*i*_ harshly attacked the senator
                                                         admitted the error.
b. The reporter_*i*_ who_*i*_ the senator harshly attacked ____*i*_
                                                         admitted the error.

Numerous studies have shown that, though (8a) and (8b) are grammatical and comprehensible, there are certain asymmetries regarding the ease (or lack thereof) with which speakers process these kinds of relative clauses. A large body of work continues to demonstrate that processing object-extracted relative clauses is more taxing, leading to increased processing times compared to subject-extracted relative clauses (see, for example, King and Just, 1991, for English; Frazier, 1987, for Dutch; Mecklinger et al., 1995, for Hungarian; Arnon, 2005, for Hebrew; Miyamoto and Nakamura, 2003, for Japanese; Kwon, 2008; Kwon et al., 2010, 2013; for Korean). Complementing the finding that object-extracted relative clauses are relatively costly to comprehend, recent work demonstrates that they are similarly costly to produce (Scontras et al., 2015).

Given the observed asymmetries in both production and comprehension costs, we might expect relative clauses to pose interesting issues for acquisition. (Recall from the previous case study the motivation for targeting psych-verbs as possible candidates for reanalysis: psych-verbs may be unstable in the native baseline, making them ideal candidates for reanalysis in heritage grammars.) For relative clauses, however, the vast literature agrees that relative clauses do not pose any special difficulties in acquisition: Children acquire these constructions by the beginning of their third year (cf. Guasti and Cardinaletti, 2003, for Romance; Flynn and Lust, 1980; Hamburger and Crain, 1982; Diessel and Tomasello, 2000, for English; Friedmann and Novogrodsky, 2004, for Hebrew; Goodluck et al., 2006, for Irish; Slobin, 1986; Özge et al., 2009, 2010, for Turkish—the list goes on and on). The contrast between psych-verbs and relative clauses is part of a larger divide in the syntax literature between so-called “A-movement” (i.e., movement to positions typically associated with arguments, like passivization), which seems to be the bane of developmental existence, and “A-bar movement” (i.e., the rest of movement, like relativization), which is acquired fairly unproblematically^4^.

(9) a. deti_i_                     [kotor-ye         ___i_                              polučili                    podarki                ot                babuški]
     children.nom.pl  rel-nom.pl                                       received                  gifts.acc.pl         from            grandma.gen
    ‘(the) children that/who received gifts from Grandma’
  b. podarki_i_              [kotor-ye        deti                               polučili                    ___i_                ot                  babuški]
     gifts.nom.pl        rel-acc.pl    children.nom.pl           received                                        from             grandma.gen
     ‘(the) gifts that the children received from Grandma’
  c. babuška_i_             [ot                   kotor-oj                      deti                           polučili          podarki         ___i_]
       grandma.nom     from                rel-gen.sg                children.nom.pl        received        gifts.acc.pl
    ‘the grandmother from whom the children received gifts’

(10) a. deti_i_                    [kotor-ye            ___i_        polučili           podarki]
       children.nom.pl  rel-nom.pl                   received         gifts.acc.pl
   b. deti                    [kotor-ye             ___i_        podarki           polučili]
       children.nom.pl rel-nom.pl                    gifts.acc.pl    received
       ‘(the) children that received (the) gifts’

Assuming that relative clauses are more firmly established in the native baseline than psych-verbs, we might expect them to be less susceptible to change in heritage grammars. If relativization does not undergo the same processes of degradation that other areas of heritage grammars do—that is, if heritage speakers and native speakers perform equally well in comprehending and producing relative clauses—we would have support for the notion that competence in relativization is independent of quantity or quality of exposure. If, however, heritage speakers do diverge from native speakers in their performance with regard to relative clauses, then the observed differences may inform the trajectory of heritage grammars.

Polinsky (2011) used a picture-matching task to investigate the relativization behavior of English-dominant heritage speakers of Russian. English and Russian are both languages where native speakers can relativize at any point in the accessibility hierarchy [see the Russian examples in (9)]. The similarity between the two systems makes the examination of relative clauses in English-dominant heritage speakers of Russian particularly compelling, as it reduces the probability of transfer. However, unlike English, Russian has rampant scrambling (see King, 1995; Bailyn, 2004). Relative clauses are no exception: in both subject- and object-extracted relative clauses, the non-extracted noun phrase may occur either pre-verbally, (10a), or post-verbally, (10b)^5^.

Given the similarities and differences between English and Russian, combined with the unique profile of abilities that characterizes heritage speakers, Polinsky's study was designed to answer two questions: first, does heritage Russian allow for the same expressivity in relativization structures, or have heritage speakers diverged from the native baseline in unnecessarily restricting themselves along the accessibility hierarchy? Second, does the presence of scrambling in the baseline Russian grammar (but not in the dominant English grammar) affect the grammar of relative clauses in the corresponding heritage language?

To answer these questions, Polinsky presented speakers with relativization structures that crossed two types of relative clause gaps (subject vs. object) with two orders of arguments in the relative clause (noun-verb vs. verb-noun). She predicted that subject-extracted relative clauses would be easier for heritage speakers to process than object-extracted structures, given the independently observed costs associated with object extraction; but she also expected the speakers would show effects of their dominant language. Specifically, Polinsky predicted that correspondences of surface order between certain Russian and English constructions would lead to differences between how heritage speakers and native speakers process scrambling within the relativization structures.

**Figure 3:**
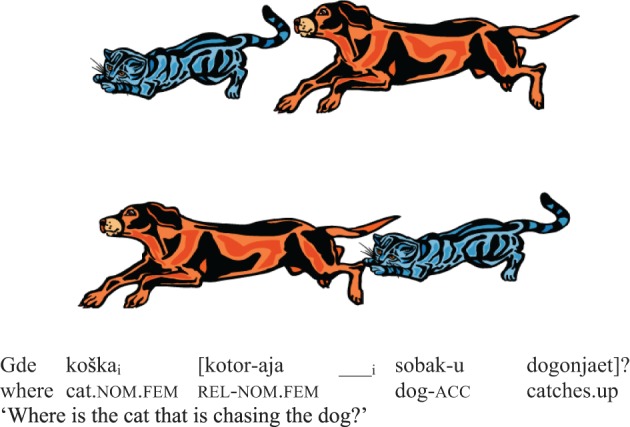
**An example item from Polinsky (2011)**.

Participants were asked to choose between two pictures as they answered an auditory question with a relative clause in it. The stimuli all featured reversible actions, for example, chasing as in Figure 3. The question varied according to whether its relative clause featured subject vs. object extraction, and whether the order of arguments in the relative clause had been scrambled.

Polinsky's monolingual speakers, both adults (*n* = 26) and children (*n* = 15), found the task almost trivial, choosing the correct picture with ceiling-level accuracy. Heritage children (*n* = 21; average age 6;0) performed equally well. The surprising case was the performance of adult heritage speakers (*n* = 29), who exhibited a stark asymmetry in their performance between subject- and object-extracted relative clauses. These participants did perform quite well in subject-extracted identification tasks, but performed at chance when asked questions involving object extraction.

Polinsky argued for attrition as the source of the difference between native and heritage adult grammars. She noted that both monolingual and heritage children performed essentially at ceiling, indicating that the adult heritage grammar could not be the result of a fossilized child language (i.e., divergent attainment), since the heritage children show perfect competence in this domain. Rather, these findings suggested that over their lifespan, the heritage speakers' competence with respect to relative clauses degraded, leaving the adult heritage speaker still capable of comprehending the easier subject-extracted relative clauses, but incapable of comprehending object-extracted relative clauses. Thus, Polinsky found evidence that relativization is not necessarily a robust area of linguistic competence: with reduced input and insufficient maintenance, competence in this area can become degraded. The observed attrition undoubtedly relates to a loss of morphological knowledge. If the heritage speakers did not process the nominative vs. accusative distinction, then they got no cue as to whether they were dealing with a subject- or object-extracted relative clause; they simply observed a clause with a transitive verb, a single overt argument, and a gap. In the absence of morphological cues, the default preference would then be to treat such a clause as a subject-extracted relative. However, this explanation alone cannot account for the comprehension of Russian relative clauses by heritage speakers, as there are also word order considerations to which we now turn.

It is natural to expect that the observed attrition may be caused by pressure from the dominant language, in this case English. If English were to blame, then relative clauses in which the internal word order mapped directly onto the word order of the analogous English sentence (i.e., relative clauses without scrambling) should have been easier for heritage speakers to process than ones in which the word orders did not match. The results of the study showed that this was not the case: heritage speakers performed equally well in identifying both subject-extracted configurations, and equally poorly in identifying both object-extracted configurations. Without any effect of scrambling on performance, we lack evidence of transfer from English. However, the absence of a scrambling effect suggests that heritage speakers were not entirely oblivious to the encoding of noun phrases, as morphology was the only cue to subject extraction in the scrambled relative clauses. Thus, Polinsky concluded that attrition in Russian heritage grammar, at least in the domain of relative clauses, is not the result of transfer. Instead, it is most likely the result of restructuring that occurs in the absence of sufficient maintenance. Ultimately, the heritage grammar is such that only subjects are accessible for relativization.

This evidence from Russian heritage grammars builds on and adds to several cross-linguistic discussions. The fact that heritage speakers performed uniformly well across subject-extracted conditions, and uniformly poorly across object-extracted conditions, regardless of word order within the relative clause, points to what has been labeled a “subject bias” observed in other syntactic environments (Keenan and Comrie, 1977; Kwon et al., 2010, 2013). Polinsky thus demonstrated that the privileged status of subjects amplifies in the heritage Russian grammar. The difference between native and heritage Russian speakers also conforms with the predictions of the accessibility hierarchy: native Russian speakers can relativize at all points on the hierarchy, whereas heritage Russian speakers can relativize at only one, the subject. This finding offers novel support to the reality of the subject as a linguistic category.

Like Pascual y Cabo (2013), this study also demonstrates the importance of comparing different age groups of heritage speakers in an effort to determine the trajectory of heritage grammars. Pascual y Cabo found that heritage adults performed better on the relevant task than children—evidence against attrition—whereas Polinsky made the same comparison but found, contrary to the expectations spelled out at the beginning of this section, that children performed better—evidence for attrition. This attrition is intriguing because it challenges the steady assumption that properties of movement (e.g., relativization), once acquired, should not be lost. It is clear, then, that a single result in one heritage group cannot be taken as evidence for a single process applying in heritage grammars across the board. Rather, in each grammatical domain and speaker population, a different combination of the factors is likely to be at play, shaping the heritage grammar.

## At the interface: Scope interpretations

Even highly advanced multilingual speakers, be they L2 learners or heritage speakers, are known to demonstrate non-target-like linguistic behavior when they have to reason simultaneously about an internal component of the grammar and an external component (e.g., discourse; Sorace, 2011, and further references therein). This so-called “Interface Hypothesis” has been studied mostly in the domain of null subject licensing, where near-native speakers, heritage speakers included, perform less consistently^6^. In an attempt to expand the range of interface phenomena under consideration, our final case study reviews experimental findings on scope interpretations in heritage grammars.

Scope interpretations bring together at least three levels of representation: syntax (expressing the structural relationship among scope-bearing elements), semantics (expressing the logical implications of this structure), and pragmatics (supporting the expressed semantics and feeding back into the choice of syntax that determines it). We might therefore expect scope calculations to diverge from the native grammar in heritage speakers, as they perform the costly operation of integrating these various levels of linguistic representation. This divergence could take one of two paths: transfer from the dominant language resulting in an otherwise uncharacteristic pattern of behavior in the heritage speaker; or, faced with two systems of relatively different complexity, the simpler system winning out in the heritage grammar. Addressing these questions makes it necessary to test multiple systems; in addition to establishing baseline data in both languages, it is desirable to test heritage speakers' knowledge of scope in both the heritage language and their dominant language.

Lee et al. (2011) take a step in this direction, trying to determine whether the grammar of scope in the heritage language could have an effect on the dominant language. The authors tested English-dominant heritage speakers of Korean on the interpretation of English negative sentences with universally quantified objects, as in (11). In English, this configuration yields ambiguity, corresponding to the scope of negation with respect to the universal quantifier.

(11) Mary didn't read all the books.
   a. *Surface scope* (¬> ∀):
   It is not the case that Mary read all the books.
   b. *Inverse scope* (∀> ¬):
   For each book, it is not the case that Mary read it.

Despite the availability of both surface and inverse interpretations for sentences like (11), speakers of English demonstrate a strong preference for surface interpretations. Presented with contexts supporting one or the other interpretation, native speakers of English accept inverse interpretations approximately 50% of the time (compared with a ceiling-level 90% acceptance rate for surface interpretations; Lee, 2009).

In Korean, similar sentences yield the opposite preference for interpretations (Han et al., 2007; O'Grady et al., 2009). Testing native speakers on sentences as in (12), Lee et al. (2011) show that surface interpretations yield near-50% acceptance rates, while inverse interpretations are accepted 90% of the time—the reverse of the English pattern.

(12) Mary-ka motwun chayk-ul anh ilk-ess-ta.
   Mary-nom all book-acc not read-pst-decl
   ‘Mary did not read all the books.’

Citing a processing explanation of these preferences from Grodner and Gibson (2005), Lee et al. suggest that differences in word order between English and Korean deliver the diverging patterns. In English, generating an inverse interpretation requires revising the initial parse, disrupting the linear operation of the processor and incurring a cost that results in a preference against the inverse, non-linear ∀ > ¬ parse. Moreover, this inverse interpretation follows unambiguously from a ready alternative utterance: *Mary didn't read any books* (cf. the “pragmatic calculus” of Lidz and Musolino, 2006). In Korean, the SOV word order has this processor first encounter the universally quantified object, then negation; using the same reasoning used for English, we correctly predict the opposite preference, namely a preference for inverse interpretations in Korean.

Moving beyond the native baseline, Lee et al. tested the interpretation preferences of English-dominant heritage speakers of Korean *in English*. Their results show that these heritage speakers deploy their Korean preferences in English: 50% acceptance rate for surface vs. 90% for inverse. Perhaps surprisingly, early exposure to Korean seemed to interfere with scope calculation in the dominant language: English. Whatever its explanation, this result nevertheless raises important questions concerning the representation of scope in both monolingual and bilingual speakers. What aspect of the dominant English grammar was affected by Korean? Unfortunately, Lee et al. did not test the scope preference of their heritage subjects in the heritage Korean grammar. Since that language was, at the time of the study, the weaker of the two in the subjects' bilingual repertoire, it is important to determine whether the scope preferences observed in monolingual Korean are still present in that language, when it is weakened by a dominant L2.

The study by Scontras et al. (2015) addresses these concerns by testing scope calculations by English-dominant heritage speakers of Mandarin in both of their languages, English and Mandarin. There is also another, more important difference between the two studies. Lee et al. demonstrate diverging preferences of scope interpretations between Korean and English in negative sentences with universally quantified objects. Crucially, speakers of each language allow both surface and inverse interpretations of these sentences, they merely prefer one interpretation over the other. However, assuming that Mandarin is a rigid surface scope language which completely disallows inverse scope in doubly-quantified sentences (an assumption which Scontras et al. test), comparing it with English, whose grammar permits inverse scope, allows for a fundamentally different comparison which more directly probes the robustness of each system as they intersect in the heritage grammar.

As in the previous case studies, the starting point is an establishment of the native speaker baseline. English sentences with more than one quantificational expression exhibit scope ambiguities. The ambiguities correspond to the relative scoping of the quantificational expressions at logical form. Various proposals deliver inverse scope; we focus on QR (May, 1977, 1985) for expository purposes and to align with discussions in previous experimental work on the topic. Under a QR approach, the surface and inverse interpretations of (13) follow from the schematic LFs in (13a) and (13b), respectively.

(13)        A shark attacked every pirate.
   a.     *Surface scope* (∃> ∀):
          There was a single shark that attacked each pirate.
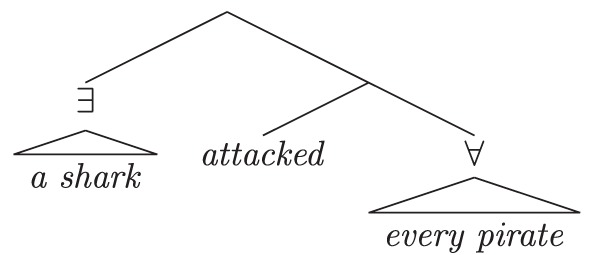
   b.     *Inverse scope* (∀> ∃):
           For each pirate, there was a (different) shark that
                                                                  attacked him.
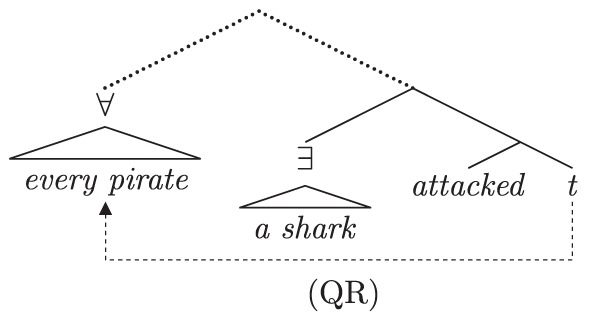

While speakers of English often accept inverse interpretations of doubly-quantified sentences, they display a reliable and robust preference for surface interpretations (cf. the preference for surface scope in negative sentences; Tunstall, 1998; Anderson, 2004). This preference holds across a variety of dependent measures (e.g., measures of grammaticality like sentence ratings and truth judgments, or measures of processing difficulty), at a range of ages. Various proposals have been put forth to explain this preference, and they all share the feature that inverse scope calculation is costly relative to surface scope. The inverse LF in (13b) involves an additional step, covert QR of the object *every pirate* above the subject *a shark*. Because of this additional operation, the inverse LF, and thus the inverse interpretation, are more complex than the surface interpretation; because it is more complex, the inverse interpretation is the less preferred of the two.

**Figure 4:**
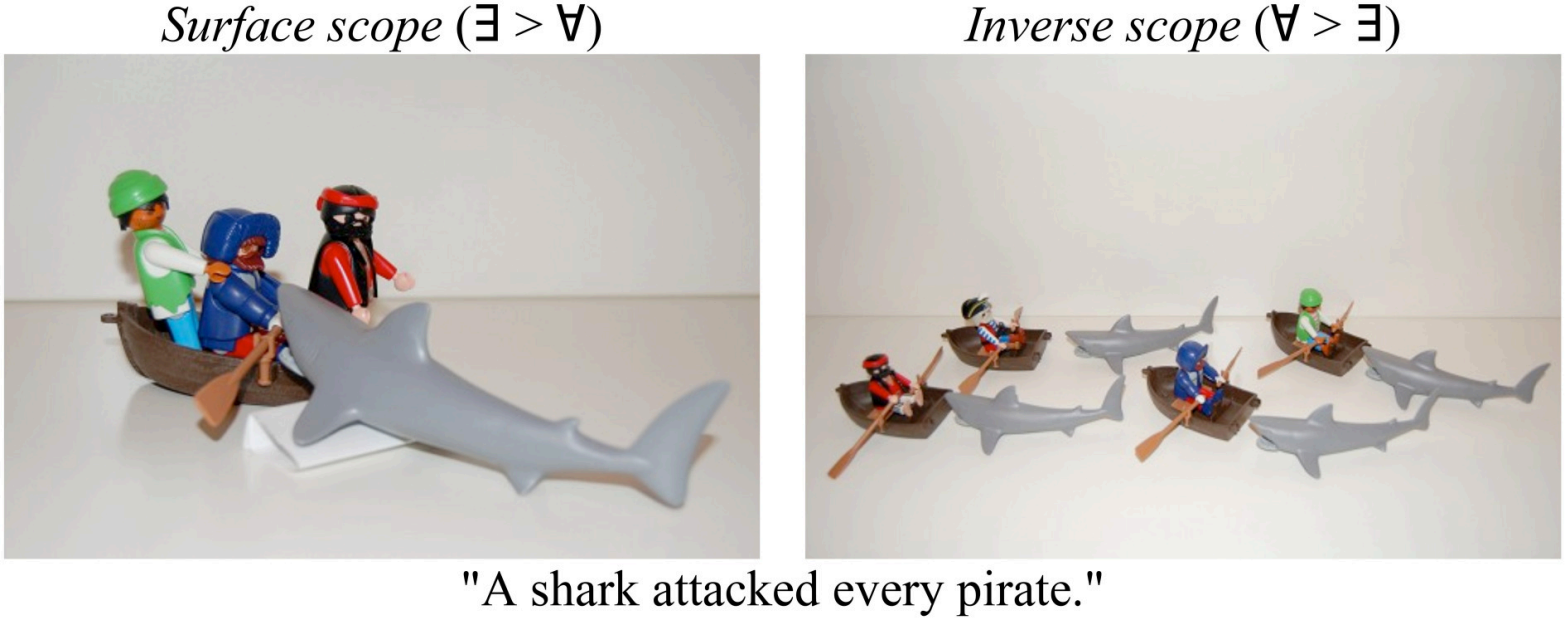
**An example item from Scontras et al. (in press)**.

**Figure 5:**
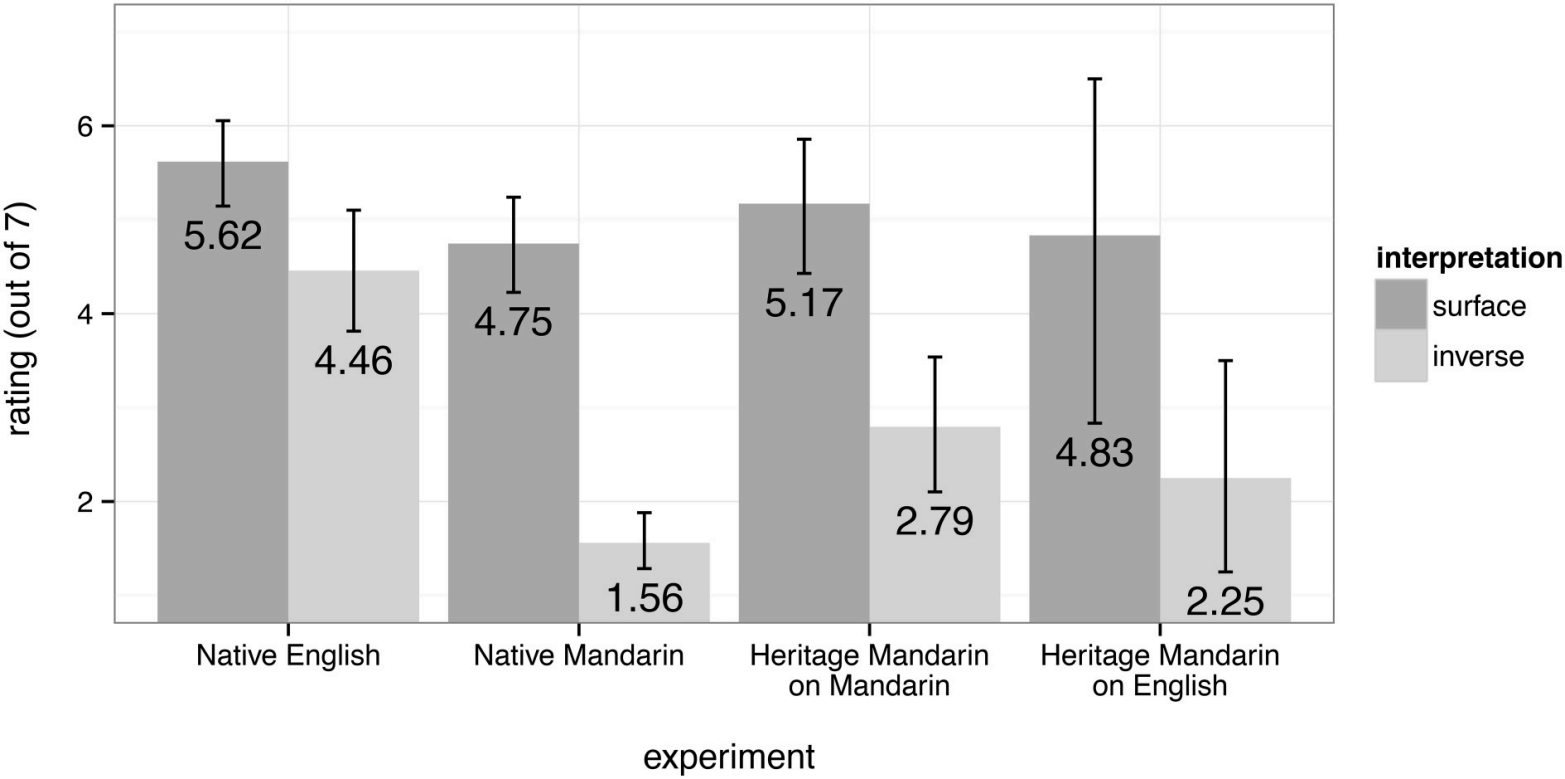
**Results from each of the four experiments from Scontras et al. (in press)**.

Scontras et al. began by demonstrating these facts about scope preferences in native English, using a scene-description-naturalness rating task. Participants (*n* = 114) were asked to judge whether the sentence they heard appropriately described a co-occurring picture using a 7-point Likert scale (1 = “completely inappropriate,” 7 = “completely appropriate”). The pictures matched either a surface (Figure 4, left) or an inverse (Figure 4, right) interpretation of the sentence^7^. Figure 5 plots average ratings by condition; error bars represent bootstrapped 95% confidence intervals drawn from 10,000 samples of the data.

As expected, native English speakers allowed inverse scope in doubly-quantified sentences. However, these inverse interpretations came at a cost, resulting in lower ratings for inverse vs. surface interpretations. Still, the average rating of 4.46 (out of 7) for inverse scope was completely in line with preceding work on English scope; in general, complex structures are associated with lower ratings, and the ratings participants assigned in this task signal that inverse scope is not impossible, but simply less preferred.

In contrast to English, the picture in Mandarin Chinese appears remarkably stark. Since Huang (1982), many linguists have arrived at or accepted the conclusion that Mandarin does not allow inverse scope in doubly-quantified sentences. This prohibition means that Mandarin translations of the English sentences we considered reportedly allow only a surface interpretation. With respect to the scenarios depicted in Figure 4, (14) should therefore be judged true only with respect to the left image.

(14) You      yi-tiao      shayu      gongji-le      mei-yi-ge              haidao.
   exist     one-clf    shark      attack-asp   every-one-clf      pirate
   ‘A/one shark attacked every pirate.’

Scontras et al. verified the claimed absence of inverse scope in Mandarin using the same sentence-picture naturalness rating task described above, this time testing native speakers of Mandarin (*n* = 53) on recorded sentences of Mandarin. Figure 5 plots the results. Consistent with the received wisdom on inverse scope in Mandarin (*pace* Zhou and Gao, 2009), subjects demonstrated a strict resistance to inverse interpretations. Put simply, Mandarin does not allow inverse scope in doubly-quantified sentences. This prohibition on inverse scope manifested as floor-level ratings, 1.56 out of a possible 7 points.

With clear baselines in hand—the availability of inverse scope in English and its absence in Mandarin—the authors then shifted their attention to the intersection of these two systems, namely English-dominant heritage speakers of Mandarin. What happens when one and the same individual presumably has access to both grammars?

Scontras et al. tested English-dominant heritage speakers of Mandarin on both the English (*n* = 11) and the Mandarin (*n* = 26) tasks described above, with the exception that the Mandarin task had instructions presented in English. The authors identified as heritage speakers those participants who learned Mandarin as their first language, but were dominant in English and lived in the United States at the time of testing. Results are plotted in Figure 5 above.

Looking first at their scope in Mandarin, the picture that emerges suggests that these English-dominant heritage speakers of Mandarin did resist inverse interpretations for doubly-quantified sentences. Their ratings for the critical inverse condition were significantly lower than the English baseline for inverse scope (2.79 heritage Mandarin vs. 4.46 native English). However, heritage speakers' ratings were higher than the native Mandarin baseline (2.79 vs. 1.56 native Mandarin). One interpretation of these facts would have the heritage participants lacking inverse scope. The higher ratings for inverse conditions (relative to native speakers) would stem instead from the “yes-bias”: heritage speakers are known to rate unacceptable/ungrammatical sequences higher than native controls (Benmamoun et al., 2013a,b; Laleko and Polinsky, 2013, in press)^8^.

Another possibility is that the heritage speakers actually found inverse interpretations in Mandarin more acceptable than did native speakers, owing to transfer from their dominant language, English. We have seen that English allows inverse scope, so perhaps this possibility has permeated the heritage Mandarin grammar. The transfer of scope shifting would be incomplete, owing to the lower ratings of inverse scope in heritage Mandarin compared to native English.

The final experiment from Scontras et al. proves crucial for teasing apart these competing hypotheses. Their results demonstrated that the English of these English-dominant heritage speakers of Mandarin does not allow inverse scope, or at least strongly resists it. These heritage speakers rated English inverse scope on average 2.25 out of a possible 7 points, a far cry from the 4.46/7 rating observed in the native English baseline. Given the observed lack of inverse scope in the English of English-dominant heritage speakers of Mandarin, it is unlikely that the intermediate ratings observed for heritage speakers tested in Mandarin stems from any transfer from a scope-allowing grammar. In fact, it would appear that these heritage speakers lack inverse scope in both their dominant English and their heritage Mandarin grammars.

By testing the robustness of the prohibition on inverse scope, the authors seem to have also tested the robustness of its permission: in the heritage speakers, even English lacked inverse scope. Could it be that the lack of inverse scope transfers from Mandarin to English in heritage speakers? Or might the relative expense of computing inverse scope, compounded with its reliance on a complex interaction between syntax, semantics, and pragmatics, render these interpretations too costly? We lack solid data to settle this question once and for all, but the authors present preliminary evidence from one last population which sheds some light on its answer: heritage speakers of English dominant in a language that prohibits inverse scope.

Given the global status of English and the prevalence of English-speaking communities, tracking down heritage speakers of English is not a trivial task. The target population for the present study is made more elusive by the requirement that these heritage speakers be dominant in a language that lacks inverse scope. Scontras et al. tested four Japanese-dominant heritage speakers of English living in Japan. Using the same English materials, these heritage speakers rated the critical inverse interpretations an average of 2.13 out of a possible 7 points. Taking into account the 4.46/7 baseline observed for native English, it appears that these heritage English speakers equally lack inverse scope. To summarize: of the four populations (native vs. heritage; English vs. Mandarin) and five grammars (native English, heritage English, native Mandarin, heritage Mandarin, and the English of heritage Mandarin speakers), Scontras et al. find just one clear case of inverse scope: the native English grammar.

Could it be that each of these heritage groups lose the ability for inverse scope because the rigid scope grammar is simpler? In fact, this is precisely what Lee et al. (2011) found for English-dominant speakers with early exposure to Korean. The confluence of evidence suggests that these bilinguals prefer simpler, less ambiguous grammars for scope—a preference visible in both the weaker and the dominant language. The authors fail to find interference from a dominant language when its system is more complex than the alternative. Instead, by expanding their sights beyond native grammars of scope, the authors found additional evidence for the precarious nature of scope calculations, manifested as a consistent pressure to simplify the grammar of scope: when two systems meet, the simpler system prevails.

If this simplification story is on the right track, the finding that heritage Mandarin speakers do not allow inverse scope in either of their languages does not necessarily entail that they have a robust Mandarin grammar. A grammar with ambiguity will be more complex than one without it: such ambiguities require abandoning a one-to-one mapping between surface structures and interpretations. The heritage Mandarin speakers that were tested might therefore have been more likely to adopt a Mandarin-like system, rather than the Mandarin system, because it is simpler, avoiding the added cost of inverse scope. In this sense, the change that resulted in the systems we observe was bidirectional, affecting both the English and the Mandarin systems. This resonates with observations, made mainly with respect to phonetics and phonology, according to which both languages in a bilingual system influence each other (cf. Flege, 1995; Flege et al., 1999, 2003; and see also Godson, 2003, for similar observations pertaining to heritage language). The results from scope thus offer novel support for the bidirectional interaction between two languages under contact.

## Conclusions

The study of multilingualism has long been the intellectual property of linguistics subfields like sociolinguistics and language acquisition, and with good reason: we must understand the complexities of the multilingual experience before we can analyze its exponence in language users. With this limitation in mind, we began by considering the heterogeneity in just one sub-population of multilinguals, namely heritage speakers. With a clearer picture of the factors at play shaping the heritage grammar, we then presented case studies appropriating heritage language study into core domains of linguistic theory: the reorganization of morphosyntactic feature systems, the reanalysis of atypical argument structure, the attrition of the syntax of relativization, and the simplification of scope interpretations. In each case, we learned not just about the idiosyncrasies of the heritage grammar, but also about the native baseline and the resources and pressures at play in the development and maintenance of grammar.

We chose these case studies to highlight the breadth of heritage language research and its implications for linguistic theory, but we also chose them to evidence some useful methods in its practice. A few practical themes repeated themselves: establishment of a clear native baseline (a must for any comparison); determination of the input to heritage language acquisition by documenting the language of the parents (to locate the potential source of reanalysis and differences from the language in the homeland); determination of child heritage language behavior (to test for attrition over the lifespan); comparison of dominant and heritage language ability in the same population (to test for transfer, and its directionality). These practices help to narrow the possible explanations for observed atypical language behavior, pointing to both the trajectory and the outcome of grammatical phenomena in heritage speakers. And while these practices necessitate a good deal of time and care on the part of the researcher, we have seen that they pay off, both by answering the specific questions targeted by the given study, and by raising additional questions central to any theory of grammar. We discuss two such questions in turn.

First, we have seen in most cases that the heritage grammar is often simpler than the native baseline with respect to the phenomenon of interest. But what does it mean to be simpler? This issue is related to two large and poorly defined notions in language science: complexity and default structures. These terms often arise in the context of sentence processing, where structures are shown to be more complex, or less default, on the basis of the processing profiles they elicit. But in the case of heritage linguistics, these terms take on a deeper meaning, one related to the grammar itself. Here we diagnosed complexity on a case-by-case basis, bringing to bear independent assumptions about language processing and architecture in the comparison of heritage and native grammars. If complexity is something that can be measured consistently, then we might expect heritage languages to consistently exhibit reduced complexity and thus reduced expressive power compared to the native baseline.

Which brings us to the second question, one we started this paper with: what does it mean to be a native speaker in the first place? Clearly the answer involves more than having L1-like phonology, which is typical of heritage speakers (Benmamoun et al., 2013b). But can we say more? On a practical note, answering this question, or at least recognizing it, is fundamental to researchers working on *understudied* and *endangered* languages. In many cases, such work involves bilingual consultants living in a dominant speech community other than the one of interest. The profile ought to ring familiar; these consultants stand a good chance of being heritage speakers of the language of interest. It is therefore possible, if not likely, that the language that gets documented will feature phenomena that are otherwise unexpected, and may seem challenging to universal principles of grammar. This issue was brought up, early on, in a seminal paper by H.-J. Sasse. He observed that differentiating native grammars “from the … situation of language decay is essential for the evaluation of data elicited from last generation speakers in a language death situation… How reliable is the speech of the last speakers [of a given community] and how much does it reveal of the original structure?” (Sasse, 1992, p. 76). As we learn more about defining properties of heritage languages, this knowledge can be used to diagnose particular phenomena that indicate divergence from the baseline even in little-documented languages. Therefore, the significance of heritage languages lies not only in and of themselves. To illustrate, heritage languages are known to avoid embedded structures (Polinsky, 2008; Benmamoun et al., 2013b); the discovery of an exotic language without embeddings—the idealization of Pirahã, to some people—will be viewed to have completely different implications if this language is used just by a handful of remaining speakers, all of them heritage.

To conclude, we believe the value of the case studies we presented and many others that we lacked the space to mention serves as a signal that the need for myopathy in linguistic theorizing has left us. The time has come to embrace multilingualism; here we have proposed a specific way to do so: studying heritage languages. If nothing else, the reality that heritage speakers are everywhere multilingualism is cries out for a better understanding of their linguistic profile. More importantly, as we mentioned at the outset, the study of grammar is necessarily an indirect enterprise, achieved by studying the behavior of speakers. Why should we not help ourselves to as many speaker populations as possible, especially when a population presents novel data and new possibilities for asking and answering questions old and new? By approaching grammar from various entry points, we stand a better chance of moving our theories from the (specific) language-centric to the (general) Language-centric, the original aim of the Chomskyan enterprise.

### Conflict of interest statement

The authors declare that the research was conducted in the absence of any commercial or financial relationships that could be construed as a potential conflict of interest.

## Footnotes

^1^Until recently, the vast majority of studies on language attrition were conducted with elderly adults (Levine, 2001; Schmid, 2011), who attained full linguistic competence before attrition began and who may also show independent aging effects.

^2^For other considerations, both empirical and theoretical, that have gone into the debate about bundling vs. split models, see Alexiadou (2004), Kramer (2014), and Ritter (1993).

^3^Here and below, the gender/number of the head noun appears first, in boldface.

^4^More generally, the vulnerability of Spanish psych-verbs reflects difficulties in the acquisition of syntactic chains of arguments, in particular the acquisition of unaccusatives (e.g., Babyonyshev et al., 2001; Machida et al., 2004).

^5^The preverbal and postverbal positions in each type of relative clause are not totally equivalent, as they differ in terms of information structure; the right edge of the clause in Russian is strongly associated with focus (Adamec, 1966; Kovtunova, 1976; Padučeva, 1985). Studies of corpora find that these differences are reflected in the relative frequency of these types of RCs in Russian (Say, 2005; Polinsky, 2011; Levy et al., 2013).

^6^The variation in near-native competency is determined by a number of factors, among which are the age of the onset of bilingualism (see Flores, 2010, 2012), the amount of input (see Montrul, 2016), and individual differences among speakers. In our discussion here, we abstract away from these additional factors.

^7^The experimental pictures were taken from Benjamin Bruening's Scope Fieldwork Project: http://udel.edu/~bruening/scopeproject/scopeproject.html.

^8^Second-language learners show a similar reluctance to reject clear grammatical violations. In their case, the lack of confidence can be attributed to their lack of implicit knowledge about many of the grammatical factors in play (Ellis, 2005, pp. 167–168).
